# Voltage‐Reconfigurable Magneto‐Ionic Nanolayers in Dot Arrays for Probabilistic, Materials‐Engineered Security Primitives

**DOI:** 10.1002/advs.76814

**Published:** 2026-07-31

**Authors:** Irena Spasojevic, Federica Celegato, Alessandro Magni, Paola Tiberto, Jordi Sort

**Affiliations:** ^1^ Departament de Física Universitat Autònoma de Barcelona (UAB) Bellaterra Spain; ^2^ Advanced Materials and Life Science Divisions Istituto Nazionale di Ricerca Metrologica (INRiM) Torino Italy; ^3^ Catalan Institute of Nanoscience and Nanotechnology (ICN2) BIST & CSIC, Campus UAB Bellaterra Spain; ^4^ Institució Catalana de Recerca i Estudis Avançats (ICREA) Barcelona Spain

**Keywords:** data security, magneto‐ionics, physical unclonable functions, probabilistic inference, security‐by‐materials‐design, true random number generators

## Abstract

The Big Data revolution demands advanced security solutions that are energy‐efficient, scalable, and resistant to emerging threats. Conventional encryption, based on algorithmic complexity, is resource‐intensive and increasingly vulnerable. To safeguard sensitive information, it is essential to develop innovative anti‐hacking and anti‐counterfeiting technologies that provide material‐level protection embedded at the smallest length scales. Here, we present a *selective magneto‐ionic strategy for hardware‐level security* that exploits voltage‐controlled N^3–^ ion migration within pre‐defined paramagnetic FeCoN dot arrays. This enables the creation of reconfigurable sub‐15 nm ferromagnetic sublayers with deterministic or probabilistic (single‐domain↔vortex) states and voltage‐tunable probabilities. These states facilitate robust magnetic fingerprinting and constitute self‐protected primitives suitable for physical unclonable functions and in‐memory probabilistic inference, while their stochastic orientation and chirality provide a platform for true random number generation. This architecture combines tamper resistance, low power consumption, and scalability, representing a significant leap toward next‐generation hardware security rooted in ion‐spin control at the nanoscale.

## Graphical abstract

A magneto‐ionic security‐by‐materials‐design strategy exploits voltage‐driven N^3–^ migration in selectively contacted FeCoN dots, creating reconfigurable sub‐15 nm ferromagnetic sublayers with deterministic and/or probabilistic (single‐domain↔vortex) states, voltage‐tunable probabilities and fully stochastic orientation/chirality. These states function as self‐protected primitives for physical unclonable functions, true random number generation, and in‐memory probabilistic inference, establishing a new hardware‐security paradigm rooted in emergent nanoscale magneto‐ionic effects.

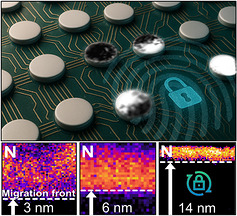

## 1 Introduction

Digital technologies are rapidly reshaping society, with data creation set to skyrocket to 221 zB by 2026. Managing “Big Data” presents major challenges in terms of processing speed, volume capacity, energy efficiency, and security. With over 422 million records breached in 2024 [1], protecting data from unauthorized access is critical. Conventional security relies on software‐based methods (passwords, two‐factor authentication, antivirus, encryption, etc.), which generate excess data and increase power consumption [2]. Additionally, physical anti‐counterfeiting features like holograms, security threads, or watermarks are often easy to clone.

Enhanced data security can be achieved through stochastic or probabilistic hardware‐based operations, which outperform software‐generated passwords and algorithmic pseudo‐random numbers by being unclonable and adversary‐resistant. True Random Number Generators (TRNGs) and Physical Unclonable Functions (PUFs) [3, 4, 5, 6, 7] are gaining attraction for cost‐effectiveness and robust protection. TRNGs provide high‐quality random bitstreams for cryptography [8], while PUFs leverage inherent randomness for rapid authentication via challenge‐response pairs (CRPs) and secure off‐device key storage [8, 9, 10, 11]. Beyond security, randomness is pivotal in emerging computing paradigms [12]. Stochastic computing encodes data as true random number (TRN)‐based bitstreams, whereas probabilistic computing uses probabilistic bits (*p*‐bits) to process probability distributions within algorithms [8, 12]. Additionally, randomness is vital in neuromorphic systems, potentially mitigating the von Neumann bottleneck [13].

Magnetic materials offer inherent randomness through effects like magnetoresistance variations (spin dice) [14], telegraphic noise in superparamagnetic tunnel junctions [15, 16], or thermally influenced skyrmions and domain wall motion [17, 18, 19]. However, challenges remain, including Joule heating, limited reconfigurability, data occupancy, and poor probabilistic control. A promising solution is energy‐efficient electric‐field control of magnetism. Magneto‐ionic materials enable precise modulation of properties such as magnetization, anisotropy, exchange bias, or spin configurations (e.g., transitions between vortex and single‐domain (SD) states [20]) via voltage‐driven ion motion [21, 22, 23, 24, 25, 26, 27]. Magneto‐ionics also provides non‐volatility (eliminating continuous power needs), semiconductor compatibility, and high endurance.

Despite the need for nanoscale control of magnetic properties, magneto‐ionic research has mainly focused on continuous films, leaving patterned systems underexplored [20, 28, 29]. Moreover, prior studies have largely concentrated on deterministic effects, overlooking stochastic/probabilistic phenomena near critical thicknesses, such as voltage‐driven SD‐vortex transitions [20]. Furthermore, few reported experiments show uniform effects across all patterned entities, with no demonstrations of selective magneto‐ionic actuation in dot arrays.

Here we demonstrate the use of magneto‐ionics as a platform for hardware security, where stochasticity arising from ion motion fluctuations, coupled with the intrinsic multi‐state capability [20, 30, 31] of patterned nanometer‐thick magneto‐ionic elements, enables the realization of reconfigurable secret keys directly at the material level. In particular, we establish the operational principle of *magneto‐ionic hardware security primitives*, including TRNGs and PUFs. Using customized electrical circuits, selective voltage protocols drive initially paramagnetic FeCoN units into probabilistic ferromagnetic SD or vortex states, exhibiting random orientation (SD) or chirality (vortex), respectively, after AC demagnetization. Intrinsically random SD orientations and vortex chiralities are exploited for TRN generation. The generated bitstreams pass all applicable NIST tests, exhibit intra‐fractional Hamming distance (FHD_intra_) close to 0.5, and reach an average Shannon entropy of approximately 0.98 per bit, confirming high‐quality stochastic behavior suitable for hardware security applications. On the other hand, voltage‐tunable realization of probabilistic SD‐vortex states in each dot is further exploited for *p*‐bit‐based PUFs and in‐memory probabilistic inference. Unlike conventional stochastic systems with passive randomness, magneto‐ionic *p*‐bit‐based devices enable externally tunable probabilistic behavior, allowing dynamically adaptive security architectures [3]. Controlling the magnetic sublayer thickness in the sub‐15 nm range via N^3^^−^ ion migration enables reconfigurable device fingerprints, whereby the overall probability distribution of SD and vortex states can be electrically tuned while the state of each individual element remains inherently unpredictable. Collectively, these results establish a new class of reconfigurable and self‐protected magneto‐ionic hardware security primitives and demonstrate a security‐by‐materials‐design approach based on electrically tunable magneto‐ionic states.

## 2 Results and Discussion

### 2.1 Selective Magneto‐Ionic Actuation Within FeCoN Functional Units

**FIGURE 1:**
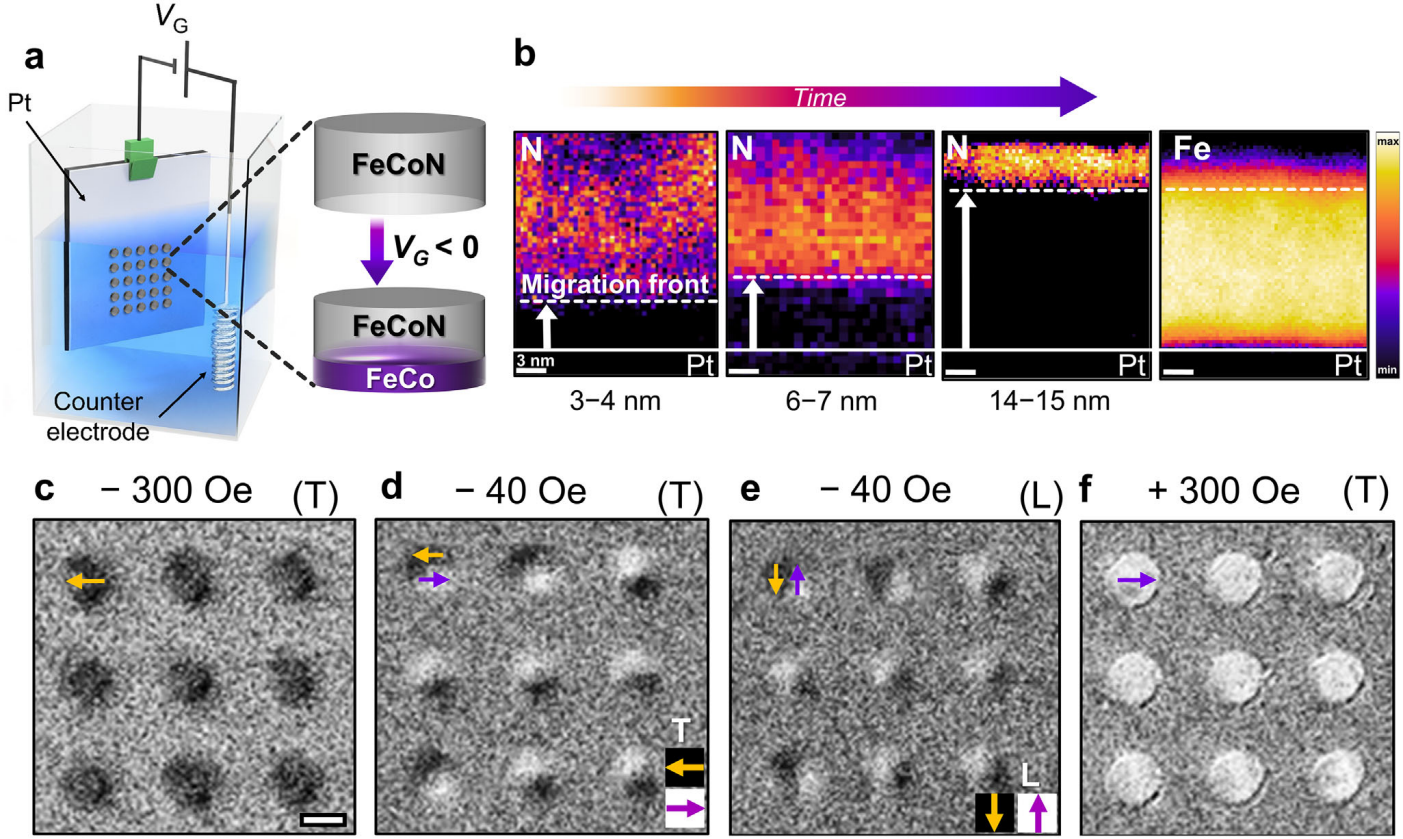
Voltage‐driven nanoscale control of magnetic layer thickness in FeCoN dots. (a) Schematic of the custom‐built electrochemical cell illustrating voltage‐driven N^3−^ ion migration within paramagnetic FeCoN dots under a negative gate voltage, *V*_G_ < 0, applied between the bottom Pt layer and the counter electrode in propylene carbonate (PC) solvent, leading to the formation of a ferromagnetic FeCo layer at the dots’ base. (b) EELS compositional mapping of cross‐sectional lamellae from FeCo(N) dots after negative voltage gating for varying durations. The EELS maps reveal the progression of a planar nitrogen migration front (white dashed line) that divides each dot into two distinct sublayers: nitrogen‐rich, paramagnetic FeCoN at the top, and nitrogen‐free, ferromagnetic FeCo at the bottom. Prolonged gating enhances nitrogen depletion and iron accumulation near the dot base, demonstrating voltage‐driven nanoscale tuning of the ferromagnetic sublayer thickness as a function of gating time. (c–f), Kerr imaging of a 3 × 3 dot array after negative gating, corresponding to the thickest magnetic layer. Images in transverse (T) geometry at −300 Oe (i.e., negative saturation) (c), −40 Oe (d), and +300 Oe (i.e., positive saturation) (f), demonstrate magnetization reversal via vortex formation in all the dots. The presence of vortex states with clockwise (CW) or counterclockwise (CCW) chirality is evident from the dark‐bright contrast in transverse T (d) and longitudinal L (e) Kerr images, indicating the curling of magnetic moments around a central core. Scale bar in (c): 2 µm.

Figure 1a illustrates the magneto‐ionic actuation mechanism in our patterned system. When 20 nm‐thick, 2 µm‐diameter FeCoN dots (Figure S1) are subjected to a negative gate voltage (*V*_G_), a planar ion migration front is generated, gradually expelling N^3^^−^ ions from the initially paramagnetic FeCoN dots and leading to the formation of ferromagnetic FeCo sublayers at their bottom. This transformation is confirmed by electron energy loss spectroscopy (EELS, Figure 1b) and Kerr imaging (Figure 1c–f). Importantly, precise control of the magnetic FeCo sublayer thickness in the sub‐15 nm range is achieved by adjusting the gating time, providing a tunable knob for tailoring the dots’ magnetic configurations. We find that the thickest FeCo sublayers within the dots exhibit a vortex magnetic state (Figure 1c–f). Although magneto‐ionic vortices were reported in our previous study [20], that work did not examine the probabilistic nature of these states, nor did the utilized setup allow voltage actuation in selected memory units. These unexplored capabilities are crucial for integrating nanoscale magneto‐ionics into complex device architectures, thereby enabling data security primitives driven by probabilistic effects.

**FIGURE 2:**
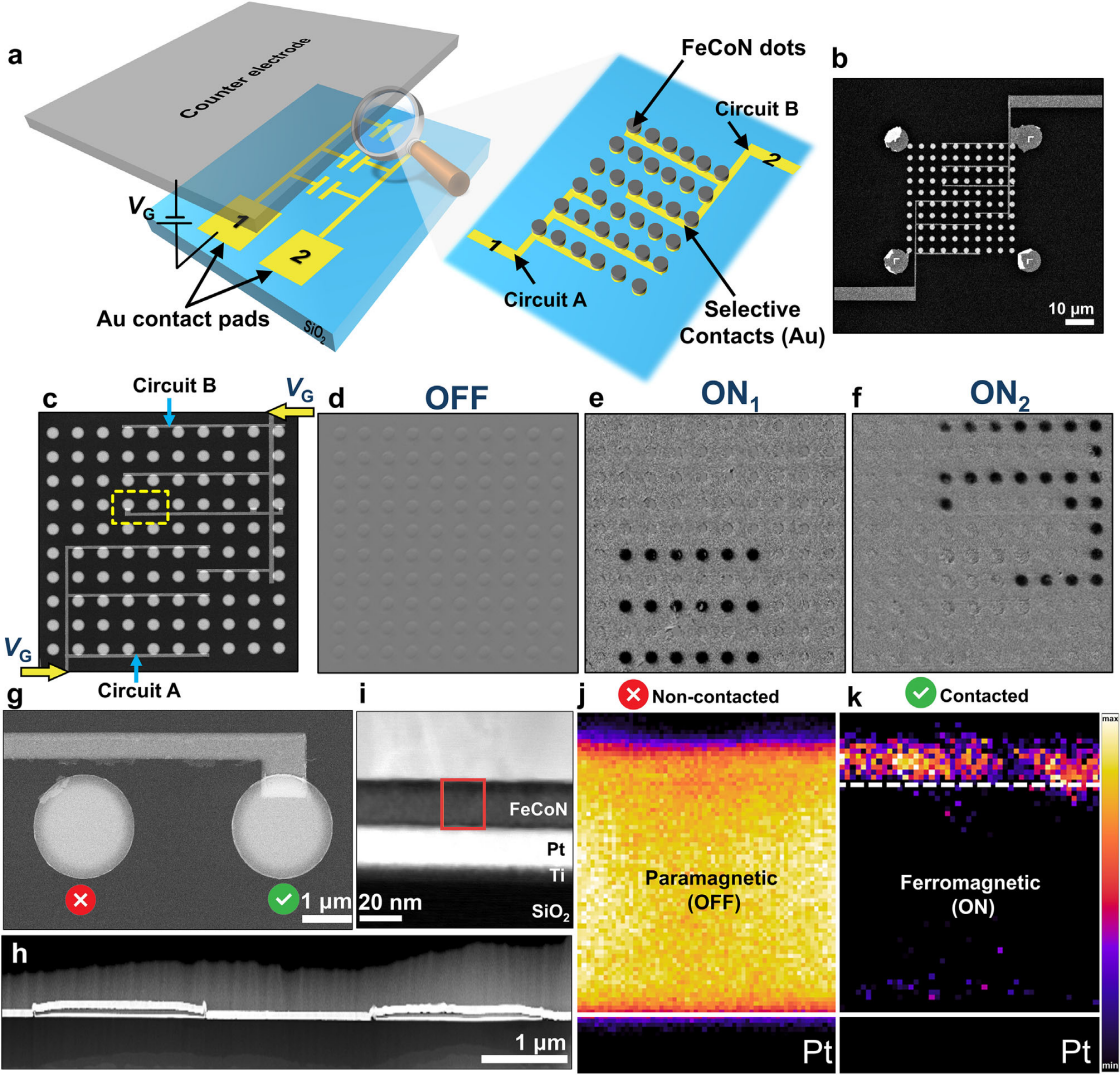
Operating principle and prototype device for selective magneto‐ionic actuation of individual circuits. (a) Schematic representation of a device, whereby gate voltage *V*_G_ < 0 between the counter electrode and either contact pad (labelled 1 and 2) selectively activates FeCoN dots within circuit A or circuit B via precisely defined lithographed contacts. A Ti(10 nm)/Pt(20 nm) bilayer deposited beneath each FeCoN(20 nm) unit ensures electrical contact, giving a total dot stack thickness of 50 nm (**Methods** and Figure S1). (b) SEM image of two nano‐sized electrical pathways interconnecting sections of a 10 × 10 dot array, along with two larger electrical contacts on each side for integration with macroscopic circuitry. (c) Details of the patterned selective contacts interconnecting the bottom left (circuit A) and top right (circuit B) part of the array. (d) Kerr imaging of the interconnected FeCoN dot array before voltage application, showing all FeCoN units in the initial paramagnetic (OFF) state. (e) Kerr image obtained under a magnetic field of −300 Oe (i.e., at saturation) after negative voltage gating of circuit A, causing initially paramagnetic FeCoN dots to become ferromagnetic (state ON_1_), while the remaining dots stay paramagnetic. (f) Application of a negative gate voltage to circuit B, which selectively activates the top‐right section of the array (state ON_2_). In both cases gate voltage *V*_G_ = −10 V was applied for *t*_1_ = 60 min. (g) SEM image of two dots, marked by a yellow rectangle in Figure 2c, where the contacted dot (green tick) underwent negative voltage treatment, while the non‐contacted dot (red cross) remains in the as‐grown paramagnetic state. (h) HAADF‐STEM image of a cross‐sectional lamella containing both dots, with a close‐up of one dot's interior in (i). (j,k) EELS nitrogen compositional mapping of non‐contacted (i.e. as grown, paramagnetic – OFF, (j)) and voltage treated (ferromagnetic – ON, (k) dot).

Figure 2a depicts a proposed device scheme for selective voltage control of magneto‐ionic units, where two circuits interconnect individual segments of the FeCoN array. Scanning electron microscopy (SEM) images of the selective gating architecture are shown in Figure 2b,c. Each magneto‐ionic unit is linked to the patterned circuitry and bridged to one of two gold contact pads (1 and 2 in Figure 2a). Kerr imaging confirms that all units were initially paramagnetic (OFF) (Figure 2d). Applying negative voltage between a selected contact pad (working electrode) and the counter electrode triggers N^3–^ ion migration exclusively in the electrically connected FeCoN dots. Representative samples and actuated circuits are detailed in **Methods**. In Sample 1, we applied *V*_G_(*t*_1_) = −10 V for *t*_1_ = 60 min to contact pad 1 to activate the bottom‐left circuit (ON_1_, circuit A), leaving the rest paramagnetic (Figure 2e), while pad 2 selectively activated the top‐right circuit (ON_2_, circuit B, Figure 2f). SEM image (Figure 2g) highlights two neighboring dots (yellow rectangle in Figure 2c), one contacted (voltage‐treated) and its adjacent non‐contacted (as‐grown). High‐angle annular dark‐field scanning transmission electron microscopy (HAADF‐STEM) images (Figure 2h,i) and nitrogen EELS mapping (Figure 2j,k) show that the non‐contacted dot retains uniform nitrogen distribution, while the gated dot exhibits nitrogen depletion and a planar migration front, fingerprint of nitrogen magneto‐ionics [20, 32]. This front enables precise control of the ferromagnetic layer thickness (in this case to ≈ 15 nm), and thus of the system's ground state (vortex vs. SD). Such control is not feasible in oxygen magneto‐ionic systems (e.g., CoO_x_, FeO_x_) [25, 28, 29], where O^2–^ migration forms irregular metallic Fe or Co clusters within the paramagnetic oxide matrix.

### 2.2 Unveiling Probabilistic and Stochastic Behavior at the Magneto‐Ionic Level

**FIGURE 3:**
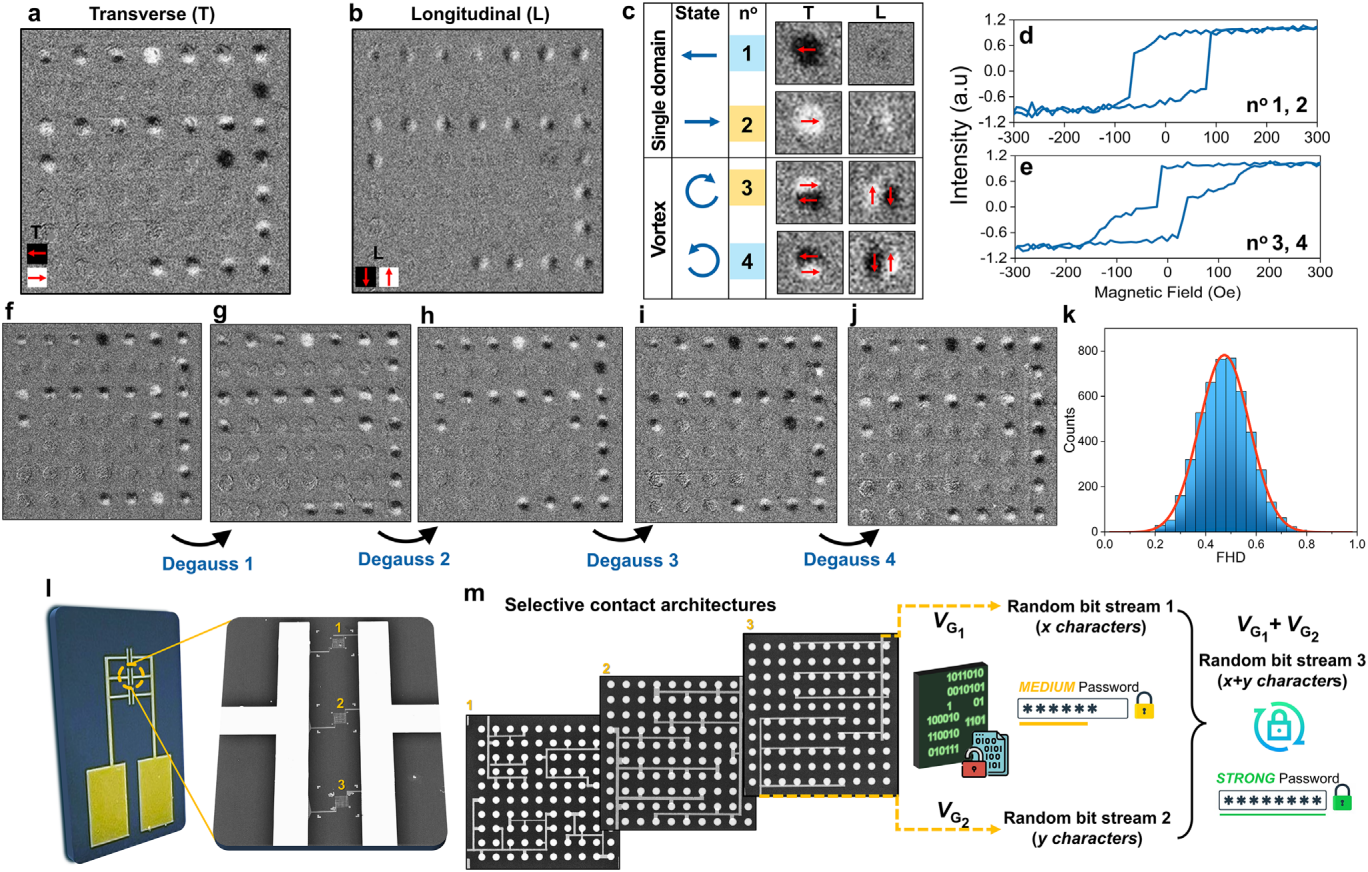
Stochastic behavior at the magneto‐ionic level – magneto‐ionic lock. Kerr imaging of the transverse T (a) and longitudinal L (b) magnetization components of dots in voltage‐treated circuit B in Sample 1 (shown in Figure 2f) after AC degaussing in the transverse direction. The latter reveals two distinct spin configurations–single‐domain (SD) or vortex–with varying directions and chiralities, giving a total of four states depicted in (c). Hysteresis loops reconstructed from Kerr imaging contrast at various applied magnetic fields for single‐domain (d) and vortex (e) states. (f–j) Transverse Kerr images obtained after successive degaussing, revealing probabilistic transitions between SD and vortex states, along with stochastic changes in SD states’ directionality and vortex chirality. (k) Intra fractional Hamming distance (FHD_intra_) between multiple degaussing cycles, considering only SD state orientation and vortex state chirality (i.e., two subclasses based on directionality obtained by grouping states 1 + 4 and 2 + 3). The red line represents the Gaussian fit of the histogram, yielding an average FHD_intra_ = 0.474, indicating fully random and uncorrelated degaussed states. Distinct selective contact architectures (l) and envisioned application of demonstrated TRNG for creating tailored‐strength *hardware* passwords (m), i.e., *magneto‐ionic locks*, where the number of password characters is controlled magneto‐ionically by activating one or more selective contacts.

An intriguing aspect of the magneto‐ionic dot array is the interplay between stochastic and probabilistic behavior, during repeated AC‐field degaussing (**Methods**). Kerr images of circuit B after transverse degaussing (Figure 3a,b) show dots adopting either a SD state oriented at 0° (state 1) or 180° (state 2), or vortex states with clockwise (CW, state 3) or counterclockwise (CCW, state 4) chirality (Figure 3c). Hysteresis loops of individual dots (Figure 3d,e) confirm these states: SD dots exhibit square‐like loops, while vortex dots display constricted loops, characteristic of vortex reversal [33]. Interestingly, consecutive degaussing cycles (Degauss 1–4) cause dynamic state evolution, leading to unexpected SD↔vortex transitions, or reversals in vortex chirality/SD orientation within individual dots (Figure 3f–j).

Assigning numerical values (1–4) to the magnetic dot states (Figure 3c) and converting images into position‐based sequences enables analysis of randomness and state correlations after repeated degaussing via FHD_intra_ (Equations (1), (2), (3), (4), **Methods**). For four states, the expected FHD_intra_ between random sequences is 0.75. In our system, FHD_intra_ is 0.505 (Figure S2), indicating probabilistic rather than entirely stochastic behavior. This is also reflected in the asymmetric outcome probabilities observed during enrolment (i.e., 100 degauss/imaging cycles): following long‐term negative voltage treatment of circuit B (*t*_1_ = 60 min), SD occurs in 8.7% of cases, while vortex dominates at 91.3%.

Considering only SD orientation and vortex chirality–i.e., grouping the four possible states into two directionality‐based subclasses–yields an average FHD_intra_ of 0.474 (Figure 3k), approaching the ideal value of 0.5 for a fully random binary system, and confirming absence of correlations between degaussed states. Therefore, voltage actuation influences SD vs. vortex probabilities while maintaining full randomness in chirality and orientation, thus unveiling a balance between control and stochasticity.

Successive degaussing further distinguishes two types of magneto‐ionic bits: (i) deterministic bits (*d*‐bits), which consistently adopt one magnetic state, and (ii) probabilistic bits (*p*‐bits), in which SD and vortex state are realized with distinct occupation probabilities. In both cases, each realization yields a stochastic magnetic directionality, manifested as a random orientation for SD states or a random chirality for vortex states. The key difference is that only *p*‐bits exhibit a statistical bias in the relative occupation of SD vs. vortex states, whereas *d*‐bits consistently favor a unique magnetic configuration. This framework clearly distinguishes between probability‐driven state selection (SD vs. vortex) and intrinsic stochasticity of orientation/chirality adopted by the resulting magnetic configuration. The interplay between these two effects determines the overall response statistics of the system. Fully stochastic SD orientations and vortex chiralities are exploited for TRNG, while probability‐weighted SD/vortex occupation in *p*‐bits forms the basis for PUF and probabilistic inference.

### 2.3 True Random Number Generator and Magneto‐Ionic Lock

The randomness in the imprinted orientation/chirality of degaussed states can be directly harnessed for TRN generation, eliminating human intervention and vulnerabilities of software‐created passwords. To quantify unpredictability and information content of binary *N*‐bit outputs, where *N* represents the number of TRN characters, we calculated the average Shannon entropy per bit $\bar{H_{i}}$ for *N* = 24 magneto‐ionically actuated dots (Equations (5) and (6), **Methods**). Given the lack of correlation in orientation/chirality (FHD_intra_ ≈ 0.5), we considered the probabilities *p_i_* of the two sub‐classes–i.e., right (CW) or left (CCW) orientation (chirality) after degaussing (Table S1). This analysis yields $\bar{H_{i}}$ = 0.97, approaching the theoretical maximum of 1, thus indicating near‐perfect randomness. The total number of possible *N*‐bit TRN sequences (*S*) is determined by total Shannon entropy (*H_total_*) and the number of active dots *N*, following *S* = 2*^Htotal = f(N)^*. For *N* = 24 this gives *S* = 1.02 × 10^7^. As shown in Figure 2e,f, the number of active dots can be reconfigured via voltage gating across several circuits, allowing the generation of TRN streams with tunable length. For instance, activating circuit A (Figure 2e) adds 18 dots with $\bar{H_{i}}$ = 0.99 (Table S2), increasing the number of available sequences to *S* = 2^41.1^ = 2.36 × 10^12^, with FHD_intra_ = 0.493 (Figure S3). The generated bitstreams pass all NIST SP 800–22 tests [34] that can be reliably evaluated at the available sequence length (see Table S3 for details).

A schematic of TRNG application for generating tailored‐strength *hardware* passwords, i.e., *magneto‐ionic locks*, is shown in Figure 3l,m, where password length is controlled magneto‐ionically. Longer passwords exponentially increase brute‐force time. In our proof‐of‐principle design, voltage can be simultaneously applied to preselected dots in nine arrays in total, with varying selective contact architectures (Figure 3l,m). Considering 100 activated dots with $\bar{H_{i}}$ = 0.98, the number of possible passwords reaches 3.17 × 10^29^. Even with an attacker guessing 1 billion passwords per second, brute‐forcing would take 10 trillion years, vastly longer than the universe's age (≈10^10^ years), rendering brute‐force attacks infeasible.

Beyond security, this approach also supports stochastic computing, where short random bitstreams enable efficient real‐time decision‐making with minimal memory usage. Thus, magneto‐ionic actuation, coupled with selective activation of specific regions within dot arrays, addresses both long‐bit security requirements and short‐bit computational efficiency.

### 2.4 Probabilistic Magneto‐Ionic Device Fingerprints: Reconfiguration and Inference

While random magnetic orientation and chirality enable TRNG operation, stochastic behavior hinders PUF‐based authentication, since physically identical samples with fully unpredictable responses under a given challenge become indistinguishable. Instead, the co‐existence of vortex and SD states in certain dots, with probabilities adjustable via voltage actuation time, offers a viable approach for magneto‐ionic PUF implementation, as demonstrated in this section.

**FIGURE 4:**
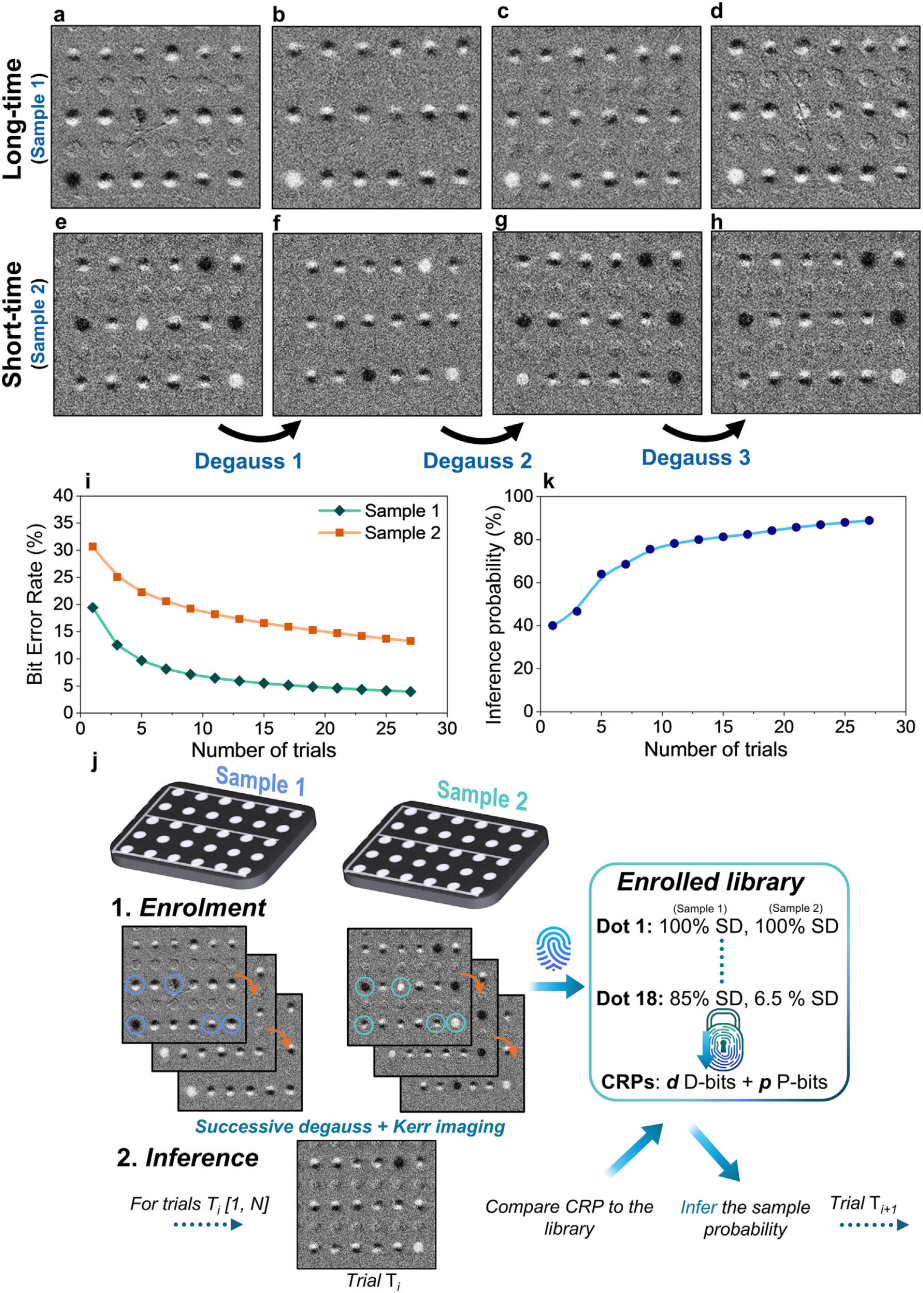
Probabilistic magneto‐ionic device fingerprints: reconfiguration and inference. Transverse Kerr images after AC degaussing and voltage treatment at *V*_G_ = −10 V of (a–d) circuit A treated for *t*_1_ = 60 min (Sample 1, “long‐time”) and (e–h) circuit of identical geometry treated for *t*_2_ = 30 min (Sample 2, “short‐time”). Shorter gating increases the number of *p*‐bits (Table 1 and Table S4) and brings their SD/vortex probabilities closer together. (i) Bit Error Rate (BER) vs. trial number for five selected *p*‐bits across both samples. (j) Probabilistic inference protocol: after each degaussing, Kerr images of five selected *p*‐bits (challenge‐response pair, CRP) from an unknown sample (e.g., Sample 1 here) are compared with “majority‐voted” states from enrolled libraries of both samples to infer whether the sample in question is Sample 1 or Sample 2. (k) Cumulatively averaging the authentication probability over 22–27 iterations ensures reliable inference with ~90% certainty.

Actuating and degaussing circuit A (Figure 4a–d) under the same conditions as circuit B yields a similar overall vortex prevalence, with SD states occurring at 10.7% probability. This happens because prolonged negative gating increases the thickness of the magneto‐ionically induced FeCo layer, stabilizing dots preferentially in a vortex state [20, 33, 35]. Tracking spin configurations of individual dots after successive degaussing allows creation of an enrolled library of SD and vortex probabilities (Table S4). In circuit A (Sample 1), eleven dots are deterministic (vortex‐only, highlighted in cyan), while seven behave as *p*‐bits (in grey), fluctuating between SD and vortex states. Considering dots’ individual state probabilities and positions, this data can be leveraged to create unique “device fingerprints” akin to PUFs, where authentication relies on measuring a device's unique physical properties through a sequence of queries (“challenges”) and their corresponding responses, forming CRPs. Here, challenges involved requesting magnetic configurations from five‐dot selections after degaussing, and the Kerr imaging gave the response bitstream. Unlike conventional PUFs with fixed responses, our *p*‐bits introduce probabilistic responses, enhancing unpredictability and CRP count. If all 18 dots in the circuit were deterministic, the number of CRPs would match the number of challenges (Equation (7)), totaling 8568. Adding 7 *p*‐bits raises this to 41 174 (4.8 times stronger PUF), while also introducing greater verification complexity.

In conventional PUFs (using only *d*‐bits), each challenge has a unique response, and the Bit Error Rate (BER) is minimal, mainly linked to experimental noise. In our method, the presence of *p*‐bits requires collecting responses across multiple trials (i.e., degaussing cycles) and classifying each bit as “vortex” or “SD” based on its most probable state determined by majority voting. Verification compares the majority‐voted response with the enrolled library, with discrepancies from *p*‐bit behavior contributing to BER. The overall BER after *T* consecutive trials is calculated using probability theory (Equations ((8), (9), (10))) by averaging error probabilities across the five‐dot positions within the CRP. Representative combinations featuring varying number of *p*‐bits are shown in Table 1 (Sample 1, Circuit A). BER progressively decreases with increasing *T*, as discrepancies statistically average out upon successive iterations. BER increases with the number of *p*‐bits in the CRPs and it also depends on the intrinsic state probabilities of each *p*‐bit. For CRPs containing 4 *d*‐bits, BER is 0.006% after 5 iterations, but BER rises to 9.6% when all five dots are *p*‐bits. However, for Sample 1, BER consistently falls below 4% after 27 iterations.

**TABLE 1:** Some representative five‐dot combinations for challenge‐response pairs in Sample 1 and Sample 2 (circuit‐type A) along with calculated Bit Error Rate (BER) and probabilistic fractional inter‐Hamming distance (pFHD_inter_). For each sample, the position of the dots in the array is identified by a number from 1 to 18. The dots are numbered following the reading direction, from left to right and top to bottom, so that dot number 1 is at the upper‐left corner, and dot number 18 is at the bottom‐right corner of circuit A in both samples. The second column indicates the number of *p*‐bits in each CRP (No. *p*‐bits), and the exact positions of the *p*‐bits in the circuit are highlighted in bold black within the CRP. Deterministic bits (*d*‐bits) that consistently adopt vortex states are highlighted in green, while those that always adopt a SD state are marked in red. The BER, calculated by comparing the device response to the enrolled library after majority voting over a specified number of trials (*T*), is shown for *T* = 1, 5, 11, and 27, with *T* indicated in the subscript. Dot combinations with calculated pFHD_inter_ close to 0.5 indicate strong uniqueness and are well‐suited for the sample differentiation via probabilistic inference.

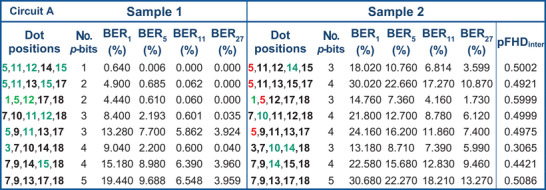

Since FeCo thickness increases with voltage actuation time (Figure 1b), further voltage exposure would drive most dots toward vortex states, eventually making them fully deterministic (and predictable), therefore reducing the number of CRPs and PUF uniqueness. To address this, actuation was shortened from *t*_1_ = 60 min (Sample 1) to *t*_2_ = 30 min (Sample 2) for circuits with identical A‐type geometry. This resulted in 9 nm‐thick ferromagnetic sublayers. Hereafter, Sample 1 and Sample 2 denote circuit A at *t*_1_ and *t*_2_ in corresponding samples. Shorter treatment time generates a new set of states (Table S4, Sample 2), increasing the average SD state probability from 10.7% to 28.2%, providing larger vortex/SD diversity (Figure 4e–h) by producing thinner FeCo sublayers within the FeCoN dots [20, 33]. Sample 2 also has more *p*‐bits (9 in total), raising CRPs to 60 858. However, BERs in Sample 2 for some selected dot combinations are higher than in Sample 1, due to the larger fraction of dots with SD probabilities in the 25%–75% range, which tend to increase BER more than dots with SD probabilities <25% or >75% (i.e., more deterministic‐like).

Although some five‐dot combinations yield appreciable BER, it will now be shown that many CRPs can be used for sample authentication through “probabilistic inference”, exploiting *p*‐bit behavior to enhance security. To identify suitable combinations, we calculate the probabilistic fractional inter‐Hamming distance (pFHD_inter_, Equations (11) and (12)) and select those with pFHD_inter_ ≈ 0.5, indicative of strong uniqueness. Examples are listed in Table 1. Typically, these combinations comprise *p*‐bits in both samples or a vortex in one sample and a *p*‐bit in the other, avoiding simultaneous vortices in both samples, which would hinder differentiation. We examine the dot combination comprising five *p*‐bits in identical positions in both samples–a configuration that maximizes initial uncertainty and BER. In this case, with just a single iteration, differentiating among the two samples is highly challenging, and incorrect guesses are likely. Despite this, BER progressively decreases with successive iterations (Figure 4i), highlighting the suitability of five *p*‐bits as robust device fingerprints.

For probabilistic inference, we selected one of the two samples (e.g., Sample 1), acquired Kerr images of the selected five *p*‐bits after each degauss (Figure 4j) and compared their states with the majority voted states in the enrolled libraries of both samples. A match with Sample 1 was confirmed if at least three *p*‐bits aligned with their enrolled states. Some tests quickly aligned with Sample 1, while in other cases, a single degauss/imaging iteration led to misclassification (40% correct inference, see example in Table S5). However, even in those cases, cumulatively averaging the authentication probability over 22–27 iterations increased inference reliability to ∼90% (Figure 4k). Since each degauss/imaging procedure takes ∼3.5 s, authentication in these intricate cases can be completed within ∼1.5 min, being considerably faster for less ambiguous responses. This also reflects an inherent trade‐off between authentication speed and confidence, which can be adjusted according to the desired level of security and the requirements of the target application.

The proposed TRNG/PUF/probabilistic inference framework offers several advantages. It is inherently robust against external magnetic fields since the vortex/SD probabilities are embedded in the material itself. Effects of magnetic field are deleted by the subsequent degauss, ensuring that probabilities remain unchanged and can only be reconfigured by voltage. This voltage‐driven reconfigurability enables post‐fabrication renewal of the device fingerprint, allowing challenge–response characteristics to be updated after deployment. Such functionality is particularly attractive from a security perspective, as it permits re‐keying following a potential security breach and increases resistance against modelling and cloning attacks that rely on static device behavior.

Additionally, the approach is wireless, requiring only AC magnetic fields and Kerr imaging, while electrical connections are needed only to set or reconfigure state probabilities, not for demagnetization or readout. The degaussing and optical readout processes are non‐destructive, causing no observable structural changes or performance degradation within the timescales of our experiments. Importantly, routine device operation does not require repeated magneto‐ionic cycling between deterministic magnetic states. Instead, state selection is performed by degaussing and optical detection, while magneto‐ionic gating is used only for initialization and, if needed, occasional reconfiguration.

Utilizing *p*‐bits instead of *d*‐bits turns probabilistic behavior into a functional advantage, enabling a large number of CRPs with minimal dot count and volume occupancy. The probabilistic nature of *p*‐bits further conceals information, as single or few Kerr images do not allow univocal identification, impeding full data reconstruction and enhancing resistance to replication or manipulation. In addition, the system provides tamper resistance by detecting unauthorized voltage application: incorrect activation of non‐addressed dots reveals intrusion and triggers an alert for reconfiguration. Moreover, *p*‐bits security measures may also help counteract brute‐force and quantum hacking attacks.

Finally, we note that we previously demonstrated [20] voltage‐driven magneto‐ionic SD↔vortex transitions in FeCoN structures with lateral dimensions approximately one order of magnitude smaller than those investigated here. While that work relied on deterministic voltage‐induced switching between SD and vortex states, the present study uses magneto‐ionic gating to tune the SD/vortex probability distribution, with the actual state selected during the degaussing process. Nevertheless, these results demonstrate that the underlying magneto‐ionic mechanism is not restricted to micrometer‐scale dimensions. The 2 µm element size was selected to enable direct Kerr microscopy visualization and statistical characterization of the magnetic‐state distributions. Therefore, the dimensions used in this study should be regarded as an experimental design choice rather than a fundamental limitation of the magneto‐ionic phenomenon or its scalability.

## 3 Conclusions

We demonstrate the selective actuation of FeCoN units within designed circuits, enabling targeted magneto‐ionic transformations that convert initially paramagnetic FeCoN into ferromagnetic vortex or SD states via voltage application. We unravel the probabilistic and stochastic nature of magneto‐ionics, demonstrating that while some dots consistently generate deterministic states (*d*‐bits) under voltage gating, others behave as *p*‐bits allowing vortex and SD states to coexist with a defined probability. Both *d*‐bits and *p*‐bits exhibit stochasticity reflected in the random orientation of SD states and the random chirality of vortex states. Voltage gating provides a means to tune the overall vortex/SD probability and thus *d*‐bit–to–*p*‐bit ratio, allowing strategic manipulation of the device fingerprints while preserving the crucial balance between control and stochasticity, essential for data security applications. Therefore, reconfigurability of the system is achieved at two levels: by selectively activating different subsets of magneto‐ionic elements through independently addressable circuits, and by electrically tuning the magnetic‐state distribution of those elements.

The random orientation and chirality of generated states can be exploited to create TRNs and hardware cryptographic keys, whereas the vortex/SD probabilities at each dot position within the array can serve as unique device identifiers. We further introduce a probabilistic inference approach that permits sample identification though an enrolment/verification process via a probabilistic PUF scheme, analogous to training/inference in neuromorphic in‐memory computing systems.

Collectively, these results establish a new class of reconfigurable and self‐protected hardware security systems based on magneto‐ionic actuation, termed magneto‐ionic hardware security primitives. Selective control of probabilistic magnetic states establishes a materials‐based route to secure authentication and anti‐counterfeiting, while also enabling multifunctional operation and parallel processing capabilities relevant to unconventional and quantum‐inspired computing architectures. Furthermore, the lithographically defined selective‐actuation architecture constitutes a solid foundation for future scaling toward larger and potentially matrix‐addressable crossbar magneto‐ionic device architectures.

## 4 Experimental Section

### 4.1 Sample and Device Preparation

Device preparation comprised three successive lithography steps, which involved patterning of FeCoN dots, lithography of microscopic selective contacts and finally preparation of macroscopic contacts. 2 µm‐diameter Ti (10 nm)/Pt (20 nm)/FeCoN (20 nm) dots, whereby the number in parenthesis denotes thickness of a given layer, were prepared by electron beam lithography (EBL) and subsequent magnetron sputtering. A single layer PMMA 4A photoresist was deposited by spinning for 60 s at 6000 rpm and baked for 5 min at 438 K on top of Si/SiO_2_ (1.5 µm) substrate. EBL was performed by using an environmental scanning electron microscope ESEM FEG Quanta 3D equipped with a NanoPattern generator system for nanolithography. Photoresist was developed using 1:3 methyl isobutyl ketone/isopropanol (MIBK/IPA) for 60 s. The bottom Ti/Pt conductive bilayer was initially deposited via DC sputtering using an AJA International ATC 2400 sputtering system in Ar atmosphere at a total pressure of 3 × 10^−3^ Torr, by channeling the corresponding targets to a DC source at 200 and 100 W power, respectively. A 20 nm thick layer of ternary nitride Fe_0.65_Co_0.35_N (FeCoN) was consecutively deposited through reactive magnetron co‐sputtering at room temperature in a mixed Ar/N_2_ atmosphere and at a total pressure of 3 × 10^−3^ Torr [32]. The Ar:N_2_ flow ratio was set at 1:1 to produce nitrogen‐rich dots with paramagnetic properties. The Fe target was powered at a constant 50 W using DC, while the Co target was powered at 55 W using an RF source. Finally, the unexposed photoresist was removed via a lift‐off process in an ultrasonic acetone bath, leaving behind Ti/Pt/FeCoN dots on top of Si/SiO_2_ substrate. In the case of the sample without selective contacts (Figure 1), FeCoN dots were grown following the same procedure on top of a continuous Ti (10 nm)/Pt (20 nm) layer, initially deposited on top of Si substrate. For selective contact architectures proposed here, microscopic contacts with a width of ≈ 500 nm used to selectively interconnect magneto‐ionic FeCoN units, were prepared by EBL following the same procedure previously described for dots, involving deposition of gold and consecutive lift‐off. Macroscopic contacts were prepared via optical lithography. First, image reversal photoresist AZ5214E was spin‐coated at 3500 rpm for 30 s onto the prefabricated microscopically interconnected Si/SiO_2_/Ti/Pt/FeCoN sample, followed by a prebake at 358 K for 60 s to ensure proper adhesion and resist activation. Using a mask aligner, a previously designed metallic mask shaped for macroscopic contacts was spatially aligned with the interconnected FeCoN arrays and exposed to UV radiation for a few seconds, followed by the reversal bake for 60 s at 358 K. After the reversal bake, the areas exposed to ultraviolet (UV) light become cross‐linked and insoluble in the developer, while the unexposed areas still behave like a normal unexposed positive photoresist. After a flood exposure (without the mask), these areas got dissolved in AZ351 developer, while the cross‐linked areas remained, therefore giving a negative image of the mask pattern. Patterning of macroscopic contacts is followed by the sputtering of gold and a final lift‐off process in acetone, giving a complete device. Representative samples and actuated circuits commented in the main text are detailed below:

**Table:** 

Sample	Circuit	Gating voltage	Gating time
**Sample 1**	Circuit A	−10 V	60 min
	Circuit B	−10 V	60 min
**Sample 2**	Circuit A	−10 V	30 min

During the magneto‐ionic actuation to activate a selective circuit of dots, a gate voltage is applied between the working electrode (contact pad) and the counter electrode (Pt wire) immersed in propylene carbonate (PC) solvent.

### 4.2 Magneto‐Optic Kerr Imaging

Kerr imaging was carried out using a high‐resolution microscope (Evico Magnetics) equipped with an oil immersion objective lens. An in‐plane magnetic field was applied using a rotatable electromagnet with split pole pieces to enhance field uniformity. This setup allows for the simultaneous acquisition of two perpendicular in‐plane components of the magnetization: transverse (parallel to the applied field) and longitudinal (perpendicular to the applied field). The sample was demagnetized from a maximum field of 300 Oe, with the field oscillating at a frequency of 23 Hz and linearly decaying over 3 s, followed by Kerr imaging. Images were acquired at 30 ms/frame (∼33 fps) with binning 2 or 120 ms/frame (∼8 fps) with binning 1. Final images were obtained by averaging 16 frames, yielding effective acquisition times of ∼0.5 s (binning 2) and ∼2 s (binning 1). In Kerr imaging, the vortex state appears as alternating dark and bright contrast dividing the dot into two distinct regions and indicating the in‐plane magnetization orientation (Figure 3c). By simultaneously obtaining transverse and longitudinal magnetization component, the chirality of the vortex can be determined. In contrast, the SD state exhibits unidirectional magnetization, resulting in a uniform magnetic contrast (black, white, or grey), depending on its orientation.

### 4.3 Electron Microscopy

Scanning electron microscopy (SEM) imaging was performed using a Zeiss Merlin SEM microscope. High‐angle annular dark‐field scanning transmission electron microscopy (HAADF‐STEM) and electron energy loss spectroscopy (EELS) were performed on a FEI TECNAI G2 F20 HRTEM/STEM microscope with a field emission gun operated at 200 kV. Cross‐sectional lamellae of the samples were cut by focused ion beam after the deposition of Pt protective layers and were subsequently placed onto a Cu TEM grid.

### 4.4 Atomic Force Microscopy

Atomic force microscopy measurements of samples’ topography were conducted in tapping mode using the MFP‐3D Origin+ Atomic Force Microscope from Asylum Research, Oxford Instruments.

### 4.5 The Intra‐Fractional Hamming Distance

FHD_intra_ after multiple degausses is determined by counting the number of differing elements between each unique pair of degaussed state sequences and then normalizing by the sequence length (i.e., the number of active dots, *N*) and the total number of unique pairs.

The Hamming distance [36] between two sequences *S_i_* and *S_j_* (each of length *N*) can be calculated as:

(1) $$HD\;\left(S_{i},S_{j}\right)=\sum_{k=1}^{N}\left(1-\delta_{S_{k}^{i},S_{k}^{j}}\right)\;$$

where $\delta_{S_{k}^{i},S_{k}^{j}}$ is the Kronecker delta function and $S_{k}^{i}$ and $S_{k}^{j}$ are the *k* ^th^ elements of the sequences *S_i_* and *S_j_*, respectively. This counts the number of positions where two sequences differ.

The number of unique pairs of degaussed images is given by the combination formula:

(2) $$Total\;pairs=\;\left(\begin{array}{c} M\\ 2 \end{array}\right)=\frac{M\left(M-1\right)}{2}$$

where *M* is the total number of acquired degaussed images.

Thus, the total sum of Hamming distances over all pairs is:

(3) $$HD_{total}=\sum_{i=1}^{M}\sum_{j=i+1}^{M}HD\left(S_{i},S_{j}\right)\;$$

The FHD_intra_ is given by:

(4) $$FHD_{intra}=\frac{2}{M\left(M-1\right)N}\sum_{i=1}^{M}\sum_{j=i+1}^{M}HD\left(S_{i},S_{j}\right)$$

### 4.6 Shannon Entropy

To quantify the average unpredictability and the information content of binary *N*‐bit outputs–where *N* represents the number of TRN characters corresponding to the number of magneto‐ionically actuated dots–we calculate the Shannon entropy for the displayed array of *N* = 24 dots as: [37]

(5) $$H_{total}=-\;\sum_{i=1}^{N}H_{i}=-\sum_{i=1}^{N}p_{i}log_{2}p_{i}+\left(1-p_{i}\right)log_{2}\left(1-p_{i}\right)$$

Where *H_i_* is entropy contribution of *i‐*th bit. Due to the excellent uncorrelation in the orientation/chirality of the degaussed states (FHD_intra_ ≈ 0.5), here we consider the probabilities *p_i_* of obtaining either a right (CW) or left (CCW) orientation (chirality) after degaussing (Table S1 and Table S2), respectively. Therefore, the total Shannon entropy of our system is expressed as:

(6) $$H_{total}=-\sum_{i=1}^{N}p_{R,CW_{i}}log_{2}\left(p_{R,CW_{i}}\right)+p_{L,CCW_{i}}log_{2}\left(p_{L,CCW_{i}}\right)$$

### 4.7 Number of Challenge‐Response Pairs

Using probabilistic theory, the number of CRPs can be determined from the following equation:

(7) $$no.\;CRP=\sum_{m=0}^{\min\left(P,k\right)}\left(\begin{array}{c} P\\ m \end{array}\right)\left(\begin{array}{c} D\\ k-m \end{array}\right)\cdot 2^{m}\;$$

where *P* is the total number of *p*‐bits in the actuated circuit, *D* is the total number of deterministic bits (so that *P* + *D* = *N*, being *N* the total number of actuated dots), *m* is the number of *p*‐bits within the *k*‐digit CRP, and *k – m* is the number of deterministic bits within the *k*‐digit CRP. Note that $\left(\begin{array}{c} P\\ m \end{array}\right)\left(\begin{array}{c} D\\ k-m \end{array}\right)$ represents the total number of challenges for a given *m*, while 2*^m^* represents the corresponding total number of responses. For *m* = 0 (all bits in the *k*‐digit CRP are deterministic, then the number of responses is 1. Also note that $\left(\begin{array}{c} P\\ m \end{array}\right)$ is a factorial number that can be calculated as:

$$\left(\begin{array}{c} P\\ m \end{array}\right)=\frac{P!}{m!(P-m)!}.$$

### 4.8 Bit Error Rate

Generally, the BER) is defined as the ratio of mismatched bits to the total number of bits in the response. In our case, to compute BER, we first calculate, for each dot, the likelihood that more than half of its trials *T* (i.e., the states obtained after each degauss) deviate from the corresponding majority‐voted enrolled state. The overall BER is then obtained by averaging these per‐dot error probabilities across all *p*‐bits within the CRP.

The probability of *k* errors in *T* trials follows a binominal distribution [38], given by:

(8) $$p\;\left(k\right)=\left(\begin{array}{c} T\\ k \end{array}\right){\left(p_{e}\right)}^{k}{\left(1-p_{e}\right)}^{T-k}$$

where *p_e_* is the probability of error in that dot (i.e., discrepancy with enrolled reference state), and (1 − *p_e_*) is the probability of success. The final error probability for that dot after majority voting, $p_{e}^{majority}$, is the sum of probabilities where the number of errors, *k*, exceeds more than a half of *T*, i.e., *k*  = ⌊*T*/2⌋  + 1. That is:

(9) $$p_{e}^{majority}=\sum_{k=\lfloor T/2\rfloor +1}^{T}p\left(k\right)$$

Finally, taking into account the error probabilities of all *N* dots, BER is given by:

(10) $$BER=\frac{\sum_{i=1}^{N}p_{e}^{majority}\left(i\right)}{N}$$

### 4.9 Inter‐Fractional Hamming Distance (FHD_inter_) in the Context of Probabilistic Inference

The uniqueness of given combinations of dots when comparing two samples can be evaluated through the calculation of the FHD_inter_. For this, the states (vortex vs. SD) can be compared dot by dot. Since the probabilities of having vortex or SD for each dot in the array are known, the FHD_inter_ can be calculated probabilistically by considering the likelihood of mismatch between corresponding dots in two samples based on their respective state [39]. For each pair of dots at position *k* in Sample 1 and Sample 2, the mismatch probability, *p*_mismatch_, can be estimated as:

(11) $$p_{mismatch,k}=p_{k}^{V1}\;p_{k}^{SD2}+p_{k}^{SD1}\;p_{k}^{V2}=p_{k}^{V1}+p_{k}^{V2}-2p_{k}^{V1}p_{k}^{V2}$$

where $p_{k}^{V1}$ and $p_{k}^{SD1}$ are the probabilities of vortex or SD state in Sample 1 at *k*‐th position, while $p_{k}^{V2}$ and $p_{k}^{SD2}$ correspond to those in Sample 2.

Then, probabilistic fractional inter‐Hamming distance (*p*FHD_inter_) is given as:

(12) $$pFHD_{inter}=\frac{1}{N}\;\sum_{k=1}^{N}p_{mismatch,k}$$

where, in our case, *N* is the total number of dots within the CRP.

## Funding

This work was supported by the European Research Council (2021‐ERC‐Advanced REMINDS Grant No. 101054687 and 2024‐ERC‐Proof of Concept ‘SECURE‐FLEXIMAG’ Grant No. 101204328); Generalitat de Catalunya (2021‐SGR‐00651); Spanish State Research Agency (PID2024‐156385OB‐I00).

## Conflicts of Interest

Authors I.S., F.C., A.M., P.T. and J.S. have filled out a patent application related to the findings in this study.

## Supporting information




**Supporting File**: advs76814‐sup‐0001‐SuppMat.docx.

## Data Availability

The authors declare that the data supporting the findings of this study are available within the paper and its supporting information files.
