# Supplementary material for: Voltage‐Reconfigurable Magneto‐Ionic Nanolayers in Dot Arrays for Probabilistic, Materials‐Engineered Security Primitives
Source: Adv Sci (Weinh). 2026 Jul 31;13(62):e76814. doi: 10.1002/advs.76814 (PMC13427368; doi:10.1002/advs.76814)
Supplement: Supplementary file 1 [file ADVS-13-e76814-s001.docx]

**Supporting Information**

**Voltage-Reconfigurable Magneto-Ionic Nanolayers in Dot Arrays for Probabilistic, Materials-Engineered Security Primitives**

*Irena Spasojevic^*^, Federica Celegato, Alessandro Magni, Paola Tiberto, Jordi Sort^*^*

I. Spasojevic, J. Sort

Departament de Física, Universitat Autònoma de Barcelona (UAB), 08193 Bellaterra, Spain

F. Celegato, A. Magni, P. Tiberto

Advanced Materials and Life Science Divisions, Istituto Nazionale di Ricerca Metrologica (INRiM), Strada delle Cacce 91, 10135 Turin, Italy

J. Sort

Catalan Institute of Nanoscience and Nanotechnology (ICN2), CSIC and BIST, 08193 Barcelona, Spain

J. Sort

Institució Catalana de Recerca i Estudis Avançats (ICREA), Pg. Lluís Companys 23, 08010 Barcelona, Spain

^*^Email: [Irena.Spasojevic@uab.cat](mailto:Irena.Spasojevic@uab.cat), [Jordi.Sort@uab.cat](mailto:Jordi.Sort@uab.cat)

**I. Atomic Force Microscopy**

**Figure S1.** 3D topography image of 2 μm dots of Ti (10 nm)/Pt (20 nm)/FeCoN (20 nm). The mentioned dot stack was grown on top of Si/SiO_2_ substrate before selective contact deposition.

**II. Intra-fractional Hamming distance considering different sources of randomness**

**Figure S2** shows calculated intra-fractional Hamming distance (FHD_intra_) for a 24-bit array (Sample 1, Circuit B) treated at *V*_G_ = – 10 V during *t*_1_ = 60 min considering four possible states: right- and left-oriented single-domain (SD) states, and clockwise (CW) and counterclockwise (CCW) vortex states. Obtained FHDᵢₙₜᵣₐ _=_ 0.505 deviates from the ideal value of 0.75 for four possible states, indicating that the system is not fully stochastic but rather exhibits a probabilistic behavior. When the analysis is limited to SD orientation and vortex chirality–two binary directionality modes, based on grouping left orientation of the SD state with CCW chirality of the vortex state, and right orientation of the SD state with CW chirality of the vortex state–the resulting average FHDᵢₙₜᵣₐ =0.474 (**Figure 3k** of the main text). This value closely aligns with the theoretical value of 0.5 expected for a random binary sequence, indicating that degaussed states are statistically uncorrelated. Actuating the complementary 18 dots (Sample 1, Circuit A) under the same conditions, and considering only SD orientation and vortex chirality, again confirms fully random behavior. The resulting FHDᵢₙₜᵣₐ of 0.493 confirms absence of correlations in orientation or chirality across degaussing cycles (**Figure S3**).

**Figure S2.** FHDᵢₙₜᵣₐ for a 24-bit array (Sample 1, Circuit B) considering four magnetic states: right- and left-oriented SD, and CW and CCW vortex. The red line indicates a Gaussian fit to the histogram, with an average FHDᵢₙₜᵣₐ = 0.505, indicating that the probability of obtaining a SD or vortex state after degaussing is unequal.

**Figure S3.** FHD_intra_ of 18-bit array (Sample 1, Circuit A). considering only the orientation of the SD states and the chirality of the vortex states (*i.e*., two direction-based subclasses). The red line is a Gaussian fit to the histogram, yielding an average FHD_intra_ = 0.493, indicating that the degaussed states, when accounting for orientation and chirality, are random and uncorrelated.

**III. Shannon entropy per bit**

We calculated the Shannon entropy for each bit in Sample 1 for two circuits: circuit B, composed of *N* = 24 magneto-ionically actuated dots, and circuit A, composed of *N* = 18 dots, as shown in **Table S1 and Table S2**, respectively. In both cases, we considered the probabilities *p_i_* of the two sub-classes after degaussing: right (clockwise) or left (counterclockwise) orientation (chirality). The resulting average Shannon entropy per bit was $\overline{H_{i}}$ = 0.99 for circuit A and $\overline{H_{i}}$ = 0.97 for circuit B, indicating a high degree of uncertainty and near-maximum randomness in the distribution of the possible states.

| **Dot position** | ***p*_R,CW_ (%)** | ***p*_L,CCW_ (%)** | **Shannon entropy/bit** |
| --- | --- | --- | --- |
| **1** | 61.29% | 38.71% | 0.963 |
| **2** | 32.26% | 67.74% | 0.907 |
| **3** | 53.23% | 46.77% | 0.997 |
| **4** | 35.48% | 64.52% | 0.938 |
| **5** | 46.77% | 53.23% | 0.997 |
| **6** | 64.52% | 35.48% | 0.938 |
| **7** | 38.71% | 61.29% | 0.963 |
| **8** | 35.48% | 64.52% | 0.938 |
| **9** | 50.00% | 50.00% | 1.000 |
| **10** | 45.16% | 54.84% | 0.993 |
| **11** | 33.87% | 66.13% | 0.924 |
| **12** | 43.55% | 56.45% | 0.988 |
| **13** | 30.65% | 69.35% | 0.889 |
| **14** | 50.00% | 50.00% | 1.000 |
| **15** | 46.77% | 53.23% | 0.997 |
| **16** | 45.16% | 54.84% | 0.993 |
| **17** | 35.48% | 64.52% | 0.938 |
| **18** | 61.29% | 38.71% | 0.963 |
| **19** | 61.29% | 38.71% | 0.963 |
| **20** | 41.94% | 58.06% | 0.981 |
| **21** | 38.71% | 61.29% | 0.963 |
| **22** | 48.39% | 51.61% | 0.999 |
| **23** | 53.23% | 46.77% | 0.997 |
| **24** | 48.39% | 51.61% | 0.999 |

**Table S1.** Shannon entropy per bit for 24 magneto-ionically actuated dots in circuit B (Sample 1). Shannon entropy was calculated by using the probabilities *p*_i_ of two subclasses: right-oriented SD and clockwise CW vortex states (*p*_R,CW_), and left-oriented SD and CCW vortex states (*p*_L,CCW_ ), observed after degaussing. The dots are numbered following the reading direction, from left to right and top to bottom, so that dot number 1 is at the upper-left corner, and dot number 24 is at the bottom-right corner of the corresponding circuit (**Figure 2f** in the main text).

| **Dot position** | ***p*_R,CW_ (%)** | ***p*_L,CCW_ (%)** | **Shannon entropy/bit** |
| --- | --- | --- | --- |
| **1** | 38.71% | 61.29% | 0.963 |
| **2** | 53.23% | 46.77% | 0.997 |
| **3** | 45.16% | 54.84% | 0.993 |
| **4** | 53.23% | 46.77% | 0.997 |
| **5** | 53.23% | 46.77% | 0.997 |
| **6** | 48.39% | 51.61% | 0.999 |
| **7** | 54.84% | 45.16% | 0.993 |
| **8** | 41.94% | 58.06% | 0.981 |
| **9** | 54.84% | 45.16% | 0.993 |
| **10** | 37.10% | 62.90% | 0.951 |
| **11** | 38.71% | 61.29% | 0.963 |
| **12** | 46.77% | 53.23% | 0.997 |
| **13** | 51.61% | 48.39% | 0.999 |
| **14** | 46.77% | 53.23% | 0.997 |
| **15** | 61.29% | 38.71% | 0.963 |
| **16** | 48.39% | 51.61% | 0.999 |
| **17** | 50.00% | 50.00% | 1.000 |
| **18** | 54.84% | 45.16% | 0.993 |

**Table S2.** Shannon entropy per bit for 18 magneto-ionically actuated dots in circuit A (Sample 1). Shannon entropy was calculated by using the probabilities *p*_i_ of two subclasses: right-oriented SD and CW vortex states (*p*_R,CW_), and left-oriented SD and CCW vortex states (*p*_L,CCW_ ), observed after degaussing. The dots are numbered following the reading direction, from left to right and top to bottom, so that dot number 1 is at the upper-left corner, and dot number 18 is at the bottom-right corner of the corresponding circuit (**Figure 2e** in the main text).

**IV. Evaluation of randomness by NIST statistical test suite**

We have performed statistical randomness testing using the NIST SP 800-22 Statistical Test Suite (STS 2.1.2) for cryptographic applications. The implementation used is an open-source version of the suite based on NIST Special Publication 800-22 (Rev. 1a).^1^

To construct the input sequences, the experimentally observed magnetic dot states were mapped into binary sequences by classifying the four possible states into two subclasses based on orientation/chirality. Each state was encoded into bit sequences of length N = 24, resulting in datasets of 99 imaging cycles. The summary of tests outcomes is provided in **Table S3.** The sequences successfully passed the Monobit, Frequency within a Block, Runs, Longest Run of Ones in a Block, Binary Matrix Rank, Discrete Fourier Transform (DFT), Non-overlapping Template Matching, Serial, and Approximate Entropy tests, indicating no detectable bias, periodicity, or short-range correlations within the available dataset. Several additional tests in the NIST suite, including Overlapping Template Matching, Maurer’s Universal Statistical, Linear Complexity, Cumulative Sums, Random Excursions, and Random Excursions Variant, were not meaningfully applicable because the available bitstreams are substantially shorter than the sequence lengths recommended for reliable evaluation by these tests. Therefore, the outcomes of these tests should not be interpreted as evidence for or against randomness.

| **#** | **Test** | **Outcome** |
| --- | --- | --- |
| **1** | monobit_test | PASS |
| **2** | frequency_within_block_test | PASS |
| **3** | runs_test | PASS |
| **4** | longest_run_ones_in_a_block_test | PASS |
| **5** | binary_matrix_rank_test | PASS |
| **6** | dft_test | PASS |
| **7** | non_overlapping_template_matching_test | PASS |
| **8** | overlapping_template_matching_test | N/A |
| **9** | maurers_universal_test | N/A |
| **10** | linear_complexity_test | N/A |
| **11** | serial_test | PASS |
| **12** | approximate_entropy_test | PASS |
| **13** | cumulative_sums_test | N/A |
| **14** | random_excursion_test | N/A |
| **15** | random_excursion_variant_test | N/A |

**Table S3.** Results of the NIST SP 800-22 statistical randomness tests applied to the generated bitstreams. Please note that are marked as *inconclusive* (N/A) due to insufficient bitstream length relative to the minimum requirements for statistically meaningful evaluation in the NIST SP 800-22 framework.

**V. Enrolled library of states’ probabilities**

Tracking the spin configurations of individual dots after successive degaussing cycles enables the creation of an enrolled library of SD and vortex state probabilities. During the enrolment process (*i.e*., the training phase), 100 degauss/imaging cycles were conducted to evaluate the probabilities of SD and vortex states. These results are presented in **Table S4** for *N* = 18 magneto-ionic elements in A-type circuit, subjected to a gate voltage of – 10 V for *t*_1_ = 60 min (Sample 1) and *t*_2_ = 30 min (Sample 2). A majority voted state is identified as the state that occurs most frequently across a set of repeated degaussed images, *i.e*. the most common state observed for a given bit.

| **Circuit A** | **Sample 1** | | **Majority voted state (SD/V)** | **Sample 2** | | **Majority voted state (SD/V)** |
| --- | --- | --- | --- | --- | --- | --- |
| **Dot position** | ***p*SD (%)** | ***p*V (%)** |  | ***p*SD (%)** | ***p*V (%)** |  |
| **1** | 0 | 100 | V | 0 | 100 | V |
| **2** | 0 | 100 | V | 0 | 100 | V |
| **3** | 0 | 100 | V | 5.9 | 94.1 | V |
| **4** | 0 | 100 | V | 0 | 100 | V |
| **5** | 0 | 100 | V | 100 | 0 | SD |
| **6** | 0 | 100 | V | 0 | 100 | V |
| **7** | 24.2 | 75.8 | V | 55 | 45 | SD |
| **8** | 0 | 100 | V | 0 | 100 | V |
| **9** | 41.9 | 58.1 | V | 11.8 | 88.2 | V |
| **10** | 11.2 | 88.8 | V | 0 | 100 | V |
| **11** | 0 | 100 | V | 27.4 | 72.6 | V |
| **12** | 0 | 100 | V | 78.4 | 21.6 | SD |
| **13** | 91.1 | 8.9 | SD | 66.6 | 33.4 | SD |
| **14** | 3.2 | 96.8 | V | 0 | 100 | V |
| **15** | 0 | 100 | V | 41.1 | 58.9 | V |
| **16** | 0 | 100 | V | 0 | 100 | V |
| **17** | 15.6 | 84.4 | V | 37.2 | 62.8 | V |
| **18** | 6.5 | 93.5 | V | 85 | 15 | SD |

**Table S4.** Enrolled library of probabilities for obtaining a SD state (*p*SD) or a vortex state (*p*V) in Sample 1 (circuit A, treated at – 10 V for 60 min) and Sample 2 (circuit A, treated at – 10 V for 30 min). Deterministic bits are highlighted in blue and probabilistic in grey color, respectively. An additional column for each sample labelled as “majority voted state” shows the most probable state of each bit after 100 degaussing/imaging cycles, determined by a majority voting procedure. The dots are numbered following the reading direction, from left to right and top to bottom, so that dot number 1 is at the upper-left corner, and dot number 18 is at the bottom-right corner of circuit A in both samples.

**VI. Inference protocol**

We implement an inference protocol to determine whether a set of input images involving five *p*-bits, corresponds to Sample 1 or Sample 2. As an example, we acquired successive images from Sample 1 after repeated degaussing (*i.e.*, trials) and, using a pre-established probability library for both samples (**Table S4**), assigned each dot to the more likely sample labelled as “1” or “2” for each trial. Final classification is performed across five *p*-bits using a majority voting scheme. In a given trial *T_i_*, the probability of the sample being identified as Sample 1 is defined as the ratio of dots classified as “1” to the total number of dots (**Table S5**). While initial iterations may yield probabilities below 50 %, occasionally leading to misclassification (see **Table S5** and **Figure 4k**), the inference process quickly corrects. After four iterations, the cumulative probability–averaging probabilities from all previous trials–exceeds 50 %, reaching 90 % after 27 iterations. In practice, this protocol can be applied to any unknown sample to verify its authenticity against one or more reference samples.

| **Dot position** | ***p*SD**  **(Sample 1)** | ***p*SD**  **(Sample 2)** | ***T*_1_** | ***T*_2_** | ***T*_3_** | ***T*_4_** | ***T*_5_** | ***T*_6_** | ***T*_7_** | ***T*_8_** | ***T*_9_** |
| --- | --- | --- | --- | --- | --- | --- | --- | --- | --- | --- | --- |
| **Dot 13** | 91.1 | 66.6 | 2 | 2 | 1 | 1 | 1 | 1 | 1 | 1 | 1 |
| **Dot 7** | 24.2 | 55 | 2 | 2 | 2 | 1 | 1 | 2 | 1 | 1 | 1 |
| **Dot 9** | 41.9 | 11.8 | 2 | 2 | 1 | 1 | 1 | 1 | 2 | 1 | 1 |
| **Dot 18** | 6.5 | 85 | 1 | 1 | 1 | 2 | 1 | 1 | 1 | 1 | 1 |
| **Dot 17** | 15.6 | 37.2 | 1 | 1 | 2 | 1 | 1 | 1 | 1 | 1 | 1 |
| **Probability per trial (%)** | | | 40 | 40 | 60 | 80 | 100 | 80 | 80 | 100 | 100 |
| **Cumulative probability (%)** | | | **40.0** |  | **46.6** |  | **64.0** |  | **68.5** |  | **75.5** |
| **Dot position** | ***p*SD**  **(Sample 1)** | ***p*SD**  **(Sample 2)** | ***T*_10_** | ***T*_11_** | ***T*_12_** | ***T*_13_** | ***T*_14_** | ***T*_15_** | ***T*_16_** | ***T*_17_** | ***T*_18_** |
| **Dot 13** | 91.1 | 66.6 | 1 | 1 | 1 | 1 | 1 | 1 | 1 | 1 | 1 |
| **Dot 7** | 24.2 | 55 | 1 | 1 | 1 | 1 | 1 | 1 | 1 | 1 | 1 |
| **Dot 9** | 41.9 | 11.8 | 2 | 1 | 1 | 1 | 2 | 1 | 1 | 1 | 1 |
| **Dot 18** | 6.5 | 85 | 1 | 1 | 1 | 1 | 1 | 1 | 2 | 1 | 1 |
| **Dot 17** | 15.6 | 37.2 | 1 | 1 | 2 | 1 | 1 | 1 | 1 | 1 | 1 |
| **Probability per trial (%)** | | | 80 | 100 | 80 | 100 | 80 | 100 | 80 | 100 | 100 |
| **Cumulative probability (%)** | | |  | **78.2** |  | **80.0** |  | **81.3** |  | **82.4** |  |
| **Dot position** | ***p*SD**  **(Sample 1)** | ***p*SD**  **(Sample 2)** | ***T*_19_** | ***T*_20_** | ***T*_21_** | ***T*_22_** | ***T*_23_** | ***T*_24_** | ***T*_25_** | ***T*_26_** | ***T*_27_** |
| **Dot 13** | 91.1 | 66.6 | 1 | 1 | 1 | 1 | 1 | 1 | 1 | 1 | 1 |
| **Dot 7** | 24.2 | 55 | 1 | 1 | 1 | 1 | 1 | 1 | 1 | 1 | 1 |
| **Dot 9** | 41.9 | 11.8 | 1 | 1 | 1 | 1 | 1 | 1 | 1 | 1 | 1 |
| **Dot 18** | 6.5 | 85 | 1 | 1 | 1 | 1 | 1 | 1 | 1 | 1 | 1 |
| **Dot 17** | 15.6 | 37.2 | 1 | 1 | 1 | 1 | 1 | 1 | 1 | 1 | 1 |
| **Probability per trial (%)** | | | 100 | 100 | 100 | 100 | 100 | 100 | 100 | 100 | 100 |
| **Cumulative probability (%)** | | | **84.2** |  | **85.7** |  | **86.9** |  | **88.0** |  | **88.9** |

**Table S5.** Demonstration of the inference protocol for Sample 1 (circuit A) across 27 successive degauss/imaging iterations using five *p*-bits. Each trial *T_i_* assigns the *p*-bits to Sample 1 or Sample 2 (1 or 2) by comparing them to the pre-established (*i.e*. enrolled) probability library (**Table** S**4**). The enrolled probabilities of the SD states (*p*SD) in Sample 1 and Sample 2 are also listed in the second and third column of this table. The probability of a vortex state is *p*V (%) = 100 – *p*SD. Initial trials (*e.g*., *T*_1_–*T*_3_) may lead to misclassification, as the cumulative probability of inferring a Sample 1, based on majority voting within each trial and averaging over all previous iterations, remains below 50 % during the first three iterations. However, cumulative inference rapidly corrects classification, surpassing 50 % confidence by iteration 4 and reaching 90 % by iteration 27.

**References**

1. Rukhin, A., Soto, J., Nechvatal, J., Smid, M., Barker, E., Leigh, S., Levenson, M., Vangel, M., Banks, D., Heckert, A., Dray, J., Vo, S. A Statistical Test Suite for Random and Pseudorandom Number Generators for Cryptographic Applications. *National Institute of Standards and Technology*, (2010).
